# Formula supplementation with human and bovine milk oligosaccharides modulates blood IgG and T-helper cell populations, and *ex vivo* LPS-stimulated cytokine production in a neonatal preclinical model

**DOI:** 10.3389/fimmu.2023.1327853

**Published:** 2023-12-20

**Authors:** Marcia H. Monaco, Mei Wang, Jonas Hauser, Jian Yan, Ryan N. Dilger, Sharon M. Donovan

**Affiliations:** ^1^Department of Food Science and Human Nutrition, University of Illinois at Urbana Champaign, Urbana, IL, United States; ^2^Brain Health Department, Nestlé Institute of Health Sciences, Société des Produits Nestlé SA, Lausanne, Switzerland; ^3^Nestlé Product Technology Center Nutrition, Vevey, Switzerland; ^4^Department of Animal Sciences, University of Illinois at Urbana Champaign, Urbana, IL, United States

**Keywords:** milk oligosaccharides, immunity, 2'-fucosyllactose (2’FL), lacto-N-neotetraose (LNnT), pig

## Abstract

**Introduction:**

Human milk contains structurally diverse oligosaccharides (HMO), which are multifunctional modulators of neonatal immune development. Our objective was to investigate formula supplemented with fucosylated (2’FL) + neutral (lacto-N-neotetraose, LNnt) oligosaccharides and/or sialylated bovine milk oligosaccharides (BMOS) on immunological outcomes.

**Methods:**

Pigs (n=46) were randomized at 48h of age to four diets: sow milk replacer formula (CON), BMOS (CON + 6.5 g/L BMOS), HMO (CON + 1.0 g/L 2’FL + 0.5 g/L LNnT), or BMOS+HMO (CON + 6.5 g/L BMOS + 1.0 g/L 2’FL + 0.5 g/L LNnT). Blood and tissues were collected on postnatal day 33 for measurement of cytokines and IgG, phenotypic identification of immune cells, and *ex vivo* lipopolysaccharide (LPS)-stimulation of immune cells.

**Results:**

Serum IgG was significantly lower in the HMO group than BMOS+HMO but did not differ from CON or BMOS. The percentage of PBMC T-helper cells was lower in BMOS+HMO than the other groups. Splenocytes from the BMOS group secreted more IL-1β when stimulated *ex vivo* with LPS compared to CON or HMO groups. For PBMCs, a statistical interaction of BMOS*HMO was observed for IL-10 secretion (p=0.037), with BMOS+HMO and HMO groups differing at p=0.1.

**Discussion:**

The addition of a mix of fucosylated and sialylated oligosaccharides to infant formula provides specific activities in the immune system that differ from formulations supplemented with one oligosaccharide structure.

## 1 Introduction

Major developmental immune milestones occur in the 12 months of life, which form the foundations of lifelong immune homeostasis (1). The immune system of the infant has been characterized by innate and adaptive systems with an ability to respond to pathogenic exposure that are distinct from those seen in adults (2, 3). More recently, the concept that the infant immune system is differentially adapted rather than less developed than the adult has been introduced (1, 4–6). Newborn infants also display a type 2 immune dominance induced during fetal life that favors an anti-inflammatory cytokine profile, although infants can mount Th1-cell mediated responsesinfants (7). Immune maturation initiates with antigenic presentation and microbial colonization (1–3). Additionally, microorganism–host interactions stimulate immune maturation in the first 3 months of life (5).

During this period of immune maturation, infants are susceptible to infection (5). Human milk provides immune and other bioactive components that modulate the development and competence of the immune system and microbiome composition (8, 9). These include the human milk oligosaccharides (HMO), which confer direct and indirect immune benefits to the infant. Some HMOs contain fucose and sialic acid moieties, which can inhibit the attachment of various microbial pathogens to cells (10). Some HMOs may interact with cell surface receptors on epithelial and immune cells (e.g., c-type lectin DC-SIGN) and influence neonatal gut and systemic immunity (11). In addition, HMO directly or indirectly modulate the gene expression and production of proteins involved in inflammation (12, 13). Lastly, HMOs have prebiotic properties that stimulate the growth of infant-type bifidobacteria and bacteroides, particularly *Bifidobacterium longum* subsp. *infantis* (14). HMO can be fermented to short chain fatty acids that may also confer immune benefit (15). Given the multifunctional activities of HMOs, there has been an increasing interest in supplementing them to infant formulas (16).

Numerous different HMO structures have been identified, and their concentration in mature milk range from 1.0-10 g/L (17, 18). In comparison, bovine milk has lower oligosaccharide concentration and structural diversity than human milk; thus, standard infant formulas have little or no detectable HMO (19). However, the availability of HMO from large-scale production has made their addition to infant formula feasible (16). Effects of 2'-fucosyllactose (2′FL) and lacto-N-neotetraose (LNnT) on immune outcomes have been reported (20) In preclinical animal models, 2′FL inhibits or prevents pathogenic microbial colonization and has modulates the immune system (21–23). LNnT is a non-fucosylated neutral milk oligosaccharide associated with higher *Bifidobacterium* abundance *in vitro* and *in vivo*, and its concentration in human milk is positively associated with 2’FL (24, 25). Clinical trials of formula supplemented with HMO have shown improvements in immune outcomes relative to control formula (26, 27). Infants fed formula containing either 0.2 or 1.0 g of 2’FL/L (along with 2.2 or 1.4 g/L of galactooligosaccharide) exhibited lower concentrations of plasma inflammatory cytokines compared to infants fed the control formula (26). In another study, infants fed formula containing 1.0 g 2’FL/L and 0.5 g LNnT/L had lower incidence of bronchitis and antipyretic and antibiotic use (27).

Until recently, pure sialylated oligosaccharides were not widely available, so preclinical and clinical studies were conducted using an oligosaccharide-enriched fraction derived from bovine milk (BMOS). Commercially available BMOS contain galactooligosaccharides, 3’-sialyllactose (3'-SL) and 6’-sialyllactose (6’-SL) (28). The addition of BMOS to infant formula resulted in the microbiome shift to resemble that of breastfed infants (29). In addition, studies indicated that enzymatic fucosylation of BMOS significantly enhanced anti-adherence properties against *Escherichia coli* O157:H7 by Caco-2 cells relative to native BMOS (30). The present study used a pre-clinical neonatal pig model to assess the immune response to a nutritionally adequate formula supplemented with BMOS and 2’FL and LNnT. The effects of BMOS and HMO on the gut microbiota and brain development and cognition outcomes are previously described (31, 32).

## 2 Materials and methods

### 2.1 Animals and sample collection

All animal care and experimental procedures were in accordance with the National Research Council Guide for Care and Use of Laboratory Animals (33) and approved by the University of Illinois at Urbana-Champaign Institutional Animal Care and Use Committee. Naturally farrowed, intact male pigs remained with the sow for two days postnatally (PND2) before being transferred and housed individually as previously described (31, 32). Briefly, pigs (n=48) were randomly assigned to four dietary treatments (n=12 per diet) based on milk replacer reconstituted at 20% w/v (ProNurse^®^ Specialty Milk Replacer, Purina Animal Nutrition, Gray Summit, MO). Diets contained milk without oligosaccharide supplementation (0 g/L, control, CON), 6.5 g/L BMOS (BMOS, Nestlé Product & Technology Center, Konolfingen, Switzerland), 1.5 g/L HMO (HMO, 1.0 g/L of 2’FL + 0.5 g/L of LNnT, Glycom, Hørsholm, Denmark), or both bovine and human milk oligosaccharides (BMOS + HMO; 6.5 g/L of BMOS+1.0 g/L of 2′FL + 0.5 g/L of LNnT). Doses of each type of oligosaccharide were chosen to replicate doses used in previous clinical studies where infants were provided formula containing 0.2 or 1 g/L 2′FL (26, 27, 34), 0.5 g/L LNnT (27), 8 g/L BMOS (35, 36), or 10 g/L BMOS (37). The analyzed oligosaccharide concentration in the formula was reported by Fleming et al. (31).

At the conclusion of the study (PND33), pigs were sedated as described by Fleming et al. (31). Blood was collected via intracardiac puncture into sodium heparin, potassium EDTA-laced, and silica-laced evacuated tubes (BD Biosciences, Mississauga, ON, Canada) for peripheral blood mononuclear cell (PBMC) isolation and plasma and serum separation, respectively. After euthanasia, the spleen, and mesenteric lymph nodes (MLN) were quickly dissected and processed for cell isolation.

### 2.2 Immune cell isolation and flow cytometry

Immune cells from PBMC, spleen and MLN were isolated for the identification of B- and T-cell population phenotypes and *ex vivo* cell stimulation assay (12). Immune cells were characterized by flow cytometry using antibodies labeled with phycoerythrin-cyanine 5 (PD-Cy5) or fluorescein isothiocyanate (FITC) dyes. T-cells were identified using mouse anti-pig CD3:PECy5 (Southern Biotech, Birmingham, AL), mouse anti-pig CD4:FITC (Clone 74-12-4, Southern Biotech) and mouse anti-pig CD8:PE (Clone 76-2-11, Southern Biotech) antibodies. The percentage of T- and B-cell populations were determined using FCS Express Cytometry 6.0 software (*De Novo* Software, Pasadena, CA). All CD3+ events were considered T-cells while subpopulation of T-cells was classified as follows; CD3+CD4+CD8-, T-helper cells; CD3+CD4-CD8+, cytotoxic T; and CD3+CD4+CD8+, double positive T-cells. CD3-CD4-CD8+ events were labeled Natural Killer (NK) cells. B-cells were identified as CD21+ and MCHII+ dual positive cells using MHCII : FITC (BioRad, Hercules, CA) and CD21:PE (Southern Biotech)-labeled antibodies.

### 2.3 *Ex vivo* stimulation of immune cells

Immune cells isolated from blood, MLN and spleen were cultured *ex vivo* to assess cytokine secretion in response to lipopolysaccharide (LPS, Millipore Sigma, St. Louis, MO) stimulation as described in Comstock et al. (12). Cells (2×10^5^ cells/well) were plated in 96-well plates and half of the cells were immediately stimulated with 2 μg/mL LPS, a TLR-4 agonist. Unstimulated and stimulated cells were cultured for 72 h at 37°C under 5% CO2, after which supernatant was separated by centrifugation and frozen until ready for analysis. Cytokine concentration in the culture supernatants were analyzed for porcine-specific cytokines (IL-1β, IL-4, IL-8, IL-10, IFN-γ, IFN-α, TNF-α) using a Luminex-based, magnetic 7-plex sandwich-ELISA technology (LSC0001M, ThermoFisher, Waltham, MA). Fold-change calculated by the mean concentration from LPS-stimulated cells divided by mean unstimulated concentration. If sample values were below the level of detection for unstimulated cells, half of the lowest concentration on the standard curve was used: IL-1β (16.05 pg/mL); IL-10 (10.5 pg/mL); TNF-α (32 pg/mL); and IL-8 (21.3 ng/mL).

### 2.4 Quantification of serum IgG. plasma LPS binding protein and major acute phase protein

The Luminex 7-plex ELISA was also used for the assessment of circulation cytokine concentration in plasma. Serum IgG (Bethyl Labs, Montgomery, TX), LPS binding protein (LPSbp; Antibodies-online, Atlanta, GA) and major acute phase protein (MAP, Cusabio, Houston, TX) concentrations were assessed using porcine-specific ELISA kits.

### 2.5 Statistical analyses

Data analysis was conducted using the MIXED procedure of SAS Enterprise Guide 7.1 (SAS Institute, Cary, NC, USA). Data were tested for normality (UNIVARIATE procedures in SAS) and arcsine-square root transformation was applied in the absence of data normal distribution. All data, except for TNF-α in conditioned media prior to LPS stimulation (unstimulated cells), were subjected to a two-way analysis of variance to assess the main effects of BMOS, HMO, and the interaction effect of BMOS and HMO. Cohort was included in the model as a random variable. For all variables, observations with a studentized residual greater than |3| were considered outliers and removed from that variable only. For unstimulated cells, data for IL-1β, IL-10, and TNF-α were not normally distributed, even after log or square root transformation. For that reason, no statistical analyses were performed for IL-1β or IL-10 secretion by unstimulated cells. IL-8 was normally distributed after log transformation and was analyzed by ANOVA. TNF-α was analyzed using an ANOVA for nonparametric data using ARTool in R (38, 39). Data were reported as means ± SEMs and statistical significance was set at p < 0.05.

## 3 Results

### 3.1 Growth and tolerance

There were no differences in growth patterns or average daily body weight gain among the pigs receiving the different diets, indicating that diets were well tolerated (31).

### 3.2 Plasma cytokines and serum LPSbp, MAP, and IgG concentrations

**Figure 1:**
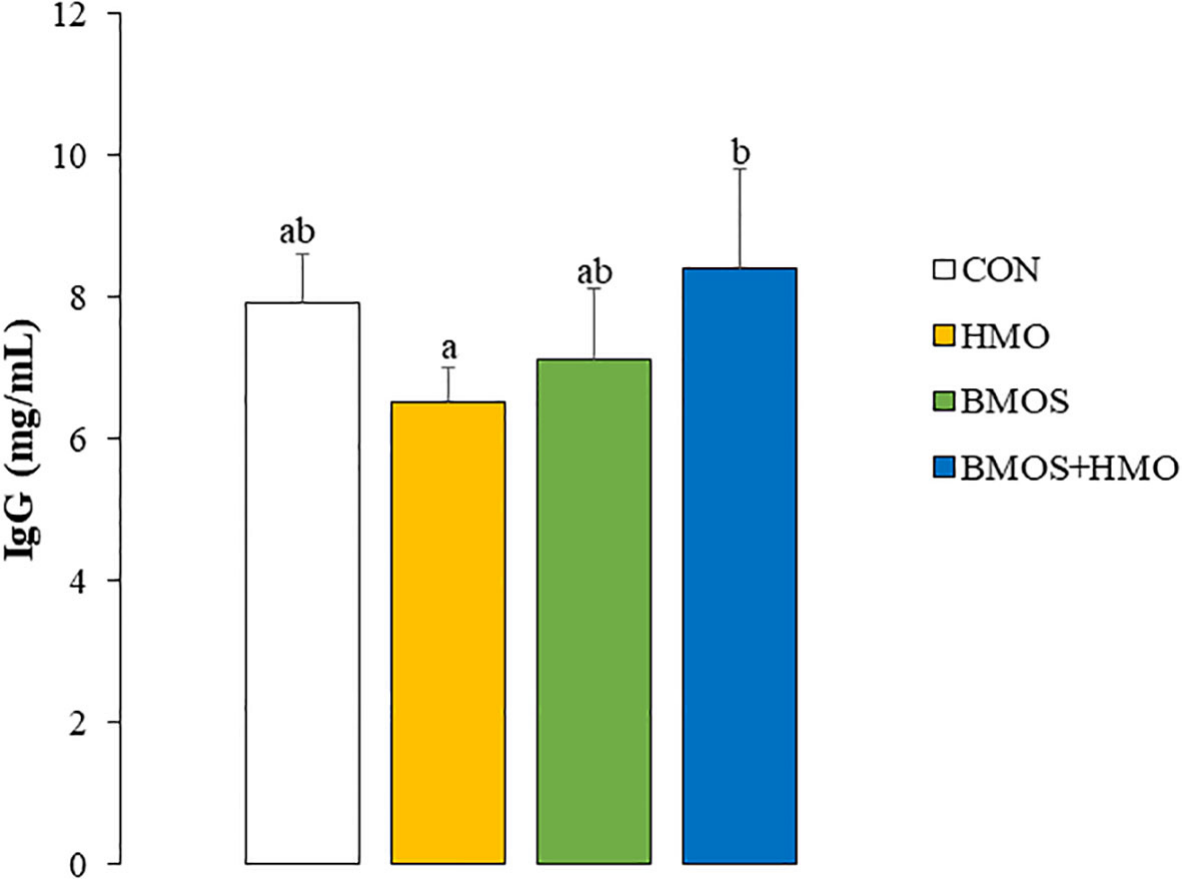
Serum IgG concentrations in 33-day-old pigs fed diets with or without BMOS and HMO. Data are expressed as means ± SEM. Different letters represent statistical differences at p<0.05.

Plasma IL-8 was not affected by dietary intervention (**Supplementary Table 1**), while the concentrations of IL-1β, IL-4, IL-10, IFN-γ, IFN-α, and TNF-α were below the level of detection (data not shown). Additionally, there was no dietary effect on serum LPSbp or MAP concentrations (**Supplementary Table 1**). There was a significant interaction between HMO and BMOS on serum IgG; IgG concentration was highest in BMOS+HMO, intermediate in CON and BMOS, and lowest in the serum of pigs fed formula with HMO (**Figure 1**).

### 3.3 Phenotypic identification of immune cells

Phenotypic distribution of T-, B-, and NK cell populations in MLN, PBMC, and spleen was assess by flow cytometry (**Table 1**). The percentage of CD3+ cells as T-helper cells was similar in PBMC and spleen but was approximately 1.5-times higher in the MLN independent of oligosaccharide exposure. The percentage of PBMC T-helper cells was significantly lower in pigs fed BMOS+HMO than those fed formula containing CON, HMO, or BMOS. Dietary treatment did not affect the percentages of double positive, cytotoxic, and NK cells in PBMC, MLN, or spleen. There was also no effect of dietary oligosaccharides on B-cell distribution in PBMC, MLN, and spleen.

**Table 1:** Phenotypic identification of immune cells in PBMC, spleen and MLN from 33-day-old pigs fed milk replacer with or without BMOS, HMO or a combination of both.

	CON (n=12)	HMO (n=12)	BMOS (n=12)	BMOS+HMO (n=10)	Statistics
PBMC	Percent of Cells*				
T-helper cells	49.4 ± 2.1 ^a^	53.7 ± 2.5 ^a^	49.9 ± 2.0 ^a^	42.7 ± 1.7 ^b^	BMOS*HMO: p=0.007
Cytotoxic T-cells	11.9 ± 1.2	12.0 ± 1.1	12.1 ± 1.1	11.9 ± 1.5	N.S.
Double positive T-cells	5.3 ± 0.6	6.2 ± 0.6	5.7 ± 0.9	5.0 ± 0.5	N.S.
Natural Killer cells	12.5 ± 2.5	10.1 ± 1.9	11.0 ± 2.5	11.5 ± 3.0	N.S.
B-cells	14.0 ± 5.6	12.3 ± 2.3	12.2 ± 2.7	11.9 ± 2.4	N.S.
MLN					
T-helper cells	72.6 ± 1.1	72.5 ± 1.0	74.9 ± 1.6	73.6 ± 2.6	N.S.
Cytotoxic T-cells	11.4 ± 0.8	12.8 ± 0.9	11.5 ± 0.9	11.0 ± 1.7	N.S.
Double positive T-cells	8.2 ± 1.1	7.3 ± 0.9	7.3 ± 0.8	8.7 ± 1.0	N.S.
Natural Killer cells	1.4 ± 0.2	1.6 ± 0.1	1.4 ± 0.1	1.4 ± 0.1	N.S.
B-cells	53 ± 3.7	61 ± 5.4	54 ± 5.2	57 ± 5.1	N.S.
Spleen					
T-helper cells	46.9 ± 2.5	47.7 ± 3.3	47.4 ± 1.3	41.9 ± 3.1	N.S.
Cytotoxic T-cells	9.9 ± 1.1	10.1 ± 1.1	10.7 ± 1.4	11.7 ± 1.3	N.S.
Double positive T-cells	3.4 ± 0.3	4.1 ± 0.6	4.6 ± 0.8	4.0 ± 0.5	N.S.
Natural Killer cells	5.6 ± 1.5	4.8 ± 1.2	4.6 ± 0.8	4.2 ± 0.7	N.S.
B-cells	41.4 ± 5.0	42.0 ± 4.5	44.4 ± 4.7	40.8 ± 7.7	N.S.

Immune cell population were characterized as: B-cells, MHCII^+^CD21^+^; Natural Killer cells, CD3^-^CD4^+^CD8; T-helper cells, CD3^+^CD4^+^CD8^+^, Cytotoxic T-cells, CD3^+^CD4^-^CD8^+^; Double positive T-cells, CD3^+^CD4^+^CD8.

*T-cell numbers are presented as % of CD3+ cells; B-cell numbers represent % of total cell count.

Data are presented as mean % ± SEM.

Different letters represent statistical differences at p<0.05; N.S. Not statistically significant.

MLN, mesenteric lymph nodes; PBMC, peripheral blood mononuclear cells; UNST, unstimulated.

### 3.4 *Ex vivo* cytokine secretion in response LPS stimulation

In conditioned media from unstimulated PBMC, cytokines were not consistently detected in all samples, ranging from 0% (IL-10) to 75-80% of samples (IL-8) (**Table 2**). There were no differences in TNF-α and IL-8 secretion by unstimulated PBMC among the treatment groups (**Table 2**).

**Table 2:** Cytokine concentrations in conditioned media of unstimulated and LPS-stimulated PBMC from 33-day-old pigs fed milk replacer with or without BMO, HMO or a combination of both.

Cytokine		CON (n=12)	HMO (n=12)	BMOS (n=12)	BMOS+HMO (n=10)	Statistics
IL-1β (pg/mL)	LPS	3329 ± 1066	2238 ± 1110	4986 ± 2528	3196 ± 600	N.S.
	UNST	74.3 (n=1)*	66.6 ± 20 (n=2)	97.8 ± 47 (n=3)	422.1 ± 261 (n=4)	
	Fold-change†	44.8	33.6	51.0	7.6	
IL-10 (pg/mL)	LPS	144 ± 17	108 ± 30	125 ± 27	166 ± 21	BMOS* HMO: p=0.037^#^
	UNST	3.5 (n=0)	3.5 (n=0)	3.5 (n=0)	3.5 (n=0)	
	Fold-change	144	108	125	166	
TNF-α (pg/mL)	LPS	1023 ± 125	626 ± 254	803 ± 170	854 ± 189	N.S.
	UNST	300 ± 145 (n=3)	304 ± 74 (n=3)	235 ± 78 (n=8)	403 ± 163 (n=4)	
	Fold-change	3.4	2.1	3.4	4.5	
IL-8 (ng/mL)	LPS	86 ± 21	49 ± 8.2	85 ± 25	64 ± 9.3	N.S.
	UNST	1.8 ± 0.6 (n=8)	1.1 ± 0.7 (n=8)	2.8 ± 1.2 (n=10)	5.7 ± 3.0 (n=8)	
	Fold-change	48	45	30	11.2	

Data are expressed, as mean ± SEM.

N.S. Not statistically significant. ^#^BMOS*HMO interaction was statistically significant, however, pairwise comparison showed a statistical difference between BMOS+HMO and HMO (p=0.1).

* Number of samples with a value above the level of detection for the ELISA.

†Fold-change was calculated by dividing the mean cytokine concentration in media of LPS-stimulated cells by the mean concentration secreted by unstimulated cell within each treatment group. If values were below the level of detection, a value representing ½ the lowest concentration on the standard curve was used: IL-1β (22.3 pg/mL); IL-10 (3.5 pg/ml); TNF-α (7.0 pg/ml); and IL-8 (31.3 pg/ml) for statistical analyses.

IL, interleukin; LPS, lipopolysaccharide; PBMC, peripheral blood mononuclear cells; TNF, tumor necrosis factor; UNST, unstimulated.

In response to LPS stimulation, secretion of a number of cytokines (IL-1β, IL-8, IL-10, TNF-α) by PBMC was numerically increased in PBMC from pigs in all treatment groups. However, due to the variation between pigs, only IL-10 reached the level of statistical significance, with an interaction of HMO and BMOS (**Table 2**). IL-10 secretion between unstimulated to LPS-stimulated PBMC increased 144-fold in the BMOS+HMO group, compared to 125-, 109-, and 94-fold in CON, BMOS, and HMO, respectively. However, differences between the groups (HMO < BMOS+HMO and CON) were only trending (p=0.11 and p=0.12, respectively) in *post hoc* analysis.

**Figure 2:**
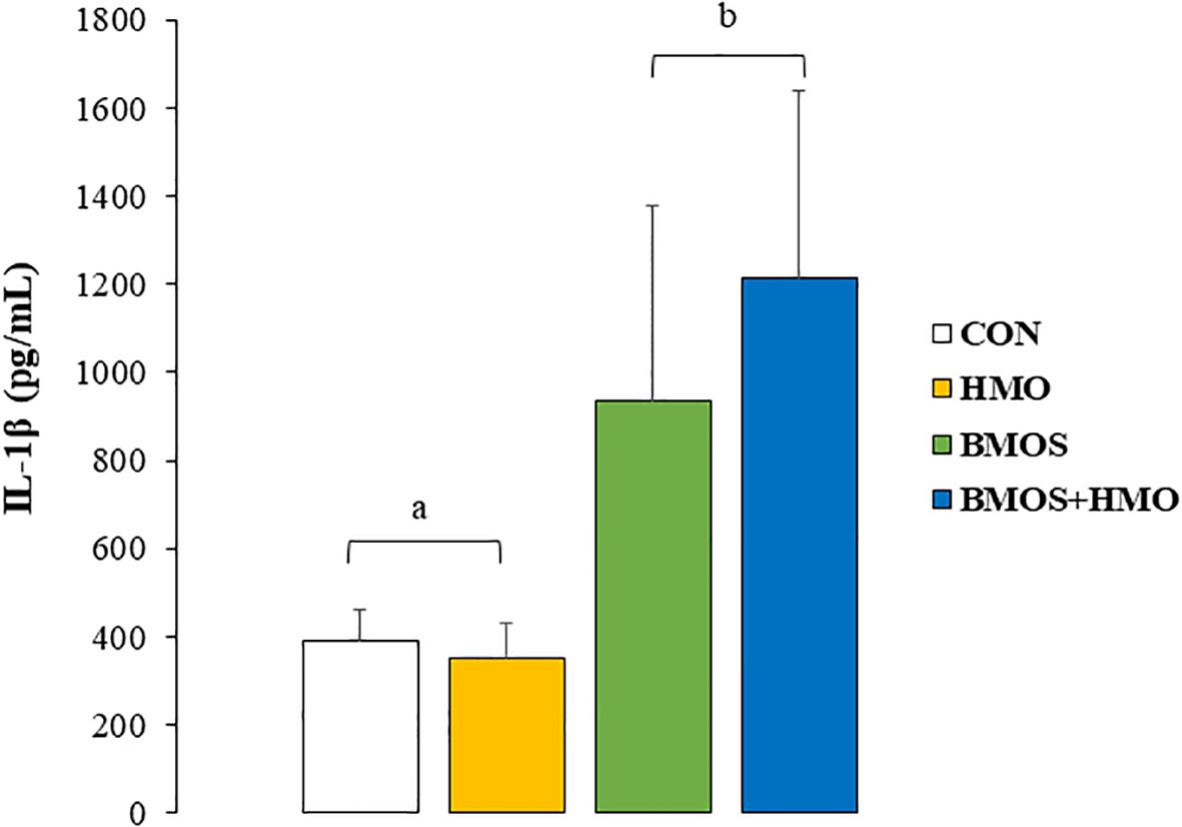
IL-1β concentrations (pg/mL) in conditioned media of by spleen cells from 33-day-old pigs fed milk replacer with or without BMO, HMO or a combination of both after incubation for 72-hours with LPS. Data are expressed as means ± SEM. Different letters represent statistical differences at p<0.05.

LPS-stimulated splenocytes secreted detectable levels of IL-1β, IL-10, and TNF-α (**Supplementary Table 2**). Statistical analysis indicated a main effect of BMOS supplementation on IL-1β secretion by LPS-stimulated splenocytes (p=0.027), with IL-1β concentration being higher in pigs fed formula containing BMOS than the other groups (**Figure 2**). Cytokine secretion by unstimulated and LPS-stimulated MLN cell media was below the detection level (data not shown).

## 4 Discussion

Existing data confirms that formula feeding leads to striking differences in the development of the immune system compared with infants fed human milk (40). Thus, there has been a concerted effort in the past 4 decades to modify the composition of infant formula, so that developmental outcomes in formula-fed infants approach those of breastfed infants (41, 42). In addition, it is well-accepted that many factors, including HMO, affect immune defense and maturation (10, 11, 15, 43). Recently, several forms of oligosaccharides have been produced in sufficient amounts to support commercialization, starting with 2′FL and LNnT (44) and, more recently, sialylated oligosaccharides (45). The present study used a pre-clinical neonatal pig model to investigate the immunomodulatory responses to oligosaccharide supplementation. The key findings indicate that formula supplementation with BMOS and HMO was well-tolerated by young pigs, allowed for healthy growth, and led to the generation of balanced responses from the arms of the immune system in an oligosaccharide-specific fashion. These findings build upon earlier publications from the same study, which demonstrated that these milk oligosaccharides not only supported normal growth (31) but influenced neurocognitive development (31) and microbiome composition (32).

In early life, the newborn heavily depends on innate immunity, as adaptive responses are not fully developed (3). Moreover, human milk enforces innate dominance. In a comparative study assessing circulating immune cell composition of breastfed and formula-fed infants from ages 1.5 to 6 months, Andersson et al. (40) reported that leukocyte counts and the frequency of B-cells in breast- and formula-fed infants were relatively similar. Lymphocyte composition differed significantly between the two groups as formula-fed infants had higher proportions of naive T-cells and T-helper (CD4+) cells but lower NK cells than breastfed infants, suggesting an immature system skewed toward adaptive immunity in formula-fed infants (40). A similar finding was observed in 10-day-old pigs, where the PBMC T-helper cell population was significantly higher in pigs fed sow-milk replacer than those fed mother’s milk (12). In the current study, pigs fed formula supplemented with BMOS+HMO had a lower percentage of the total PBMC lymphocyte population as T-helper cells compared with pigs fed CON, HMO, or BMOS, while cytotoxic and double positive T-cells were unaffected. While administration of 2’FL and LNnT or the sialic acid-enriched BMOS alone did not modulate the PBMC lymphocyte population profile, the combined effect of both BMOS and HMO on T-helper cell populations suggests that a more complex combination of oligosaccharides, such as those found in human milk, is needed to produce a T-helper phenotypic cell distribution in formula-fed animals similar to that of breastfed.

The effect of combined BMOS and HMO was also observed in the PBMC *ex vivo* experiments. IL-10 secretion by LPS-stimulated PBMC tended to be lower in the piglets fed formula with HMO than BMOS+HMO (p=0.1), although neither group differed from CON. A previous study found that 2’FL inhibited the expression of the LPS co-receptor leading to a weakened LPS-induced inflammatory response in an *Escherichia coli*-infected intestinal epithelial cell experiment (46). IL-10 is an immunomodulatory cytokine that lowers pro-inflammatory cytokine production by reducing Th1 cell, NK cell, and macrophage activities, contributing to a shift toward a more balanced Th1/Th2 (47). We have shown that *ex vivo* stimulation of pig PBMC with a mixture of HMO (comprised of 56% fucosylated and 32% sialylated oligosaccharides) resulted in higher secretion of IL-10 compared to cells exposed to individual oligosaccharides (12). It has been hypothesized that upregulation of interleukin secretion, such as IL-10, by HMO is due to their role in enhancing dendritic cell function, which impacts T-cell regulation (48, 49). These results suggest that the role of oligosaccharides on the Th1/Th2 balance is associated with exposure to more than one type of HMO. The LPS challenge in the current study sought to model a gram-negative bacterial infection, we have shown that a combination of four HMO (2′FL, LNnT, 3′-SL, 6′-SL) and sialic acid at a total concentration of 4g/L shortened the duration of rotavirus (RV) infection in piglets and upregulated the expression of IL-8 and both Th1 (IFN- γ) and Th2 (IL-10) cytokines in ileal tissue (50). Subsequent analysis of PBMCs collected prior to and 5 days post-RV infection suggested that consuming formula with HMOs affected the function of PBMCs irrespective of RV infection (22). For example, upon exposure to RV antigens *ex vivo*, PBMCs isolated from noninfected piglets fed formula with HMO had 2-fold more IFN-γ–producing cells PBMCs from formula-fed noninfected pigs (22).

Fucosylated HMO activities have been considered Th2-promoting, which leads to lower circulating levels of pro-inflammatory cytokines (43, 51), although others reported a Th1 and regulatory response *in vitro* in response to 2’FL (52). On the other hand, sialylated HMO is believed to have a multidirectional function on immunity, with both Th2-driving anti-inflammatory and Th1-shifting pro-inflammatory responses (43). We observed a significant main effect of BMOS on IL-1β production by LPS-stimulated splenocytes *ex vivo*. Spleen cells from animals fed formula supplemented with BMOS (BMOS and BMOS+HMO) secreted more IL-1β than those not receiving it (CON or HMO), suggesting a possible strengthening of Th1 response. IL-1β acts on the priming of various immune cells, such as neutrophils, dendritic cells, NK cells, macrophages, and T cells, which promotes lymphocyte maturation and up-regulates pro-inflammatory cytokines and chemokines secretion (53). In HT-29 cells, IL-1β gene expression was up-regulated these colonic epithelial cells were exposed to a mixture of HMO or BMOS but not 3’ sialyllactose alone (54). Others showed that cell stimulation with acidic oligosaccharides increased the *ex vivo* secretion of pro-inflammatory cytokines (interferon-gamma and IL-13) in human cord blood cells (55). In contrast, splenocytes isolated from mice vaccinated against influenza vaccination that were exposed to acidic oligosaccharide *ex vivo* had lower secretion of Th2 cytokines (IL-4, IL-5, and IL-10). These studies prove that mixtures predominantly containing acidic oligosaccharides favor Th1-type immunity.

A few prior studies have investigated the effect of milk oligosaccharides on humoral immunity, but those studies primarily focused on administration of a single oligosaccharide. In mice, 2’FL increased influenza vaccine-specific immunoglobulin (Ig) G1 and IgG2a by stimulating B-cell frequency and activation (23). Additionally, suckling rats receiving 0.2 g 2’FL/100 g body weight by oral gavage for 14 days had higher plasma IgG than animals dosed with vehicle solution, primarily due to a 50% increase in IgG2b (51). Coversely, Duan et al. (56) reported that weaned pigs fed a basal diet with 5.0 g of sialyllactose/kg body weight had higher serum IgG, IgA, and IgM than animals fed an unsupplemented diet. Although serum immunoglobulins decreased when the same pigs were orally challenged with enterotoxigenic *Escherichia coli* (ETEC), dietary sialyllactose tended to partially ameliorate the decline in serum IgA following the ETEC challenge (56). In the present study, supplementing BMOS+HMO resulted in higher circulating IgG concentrations relative to pigs supplemented with either HMO (p<0.05) or BMOS (p=0.1). IgG, a component of the Th1 arm of the immune system, may be derived from both the systemic and mucosal immune systems and depends on a complex milieu involving B-cells, antigen-presenting cells, and cytokines produced by T-helper cells (57). An alternative view on the secretion of IgG is the role of the microbiota on influencing humoral immunity. Studies have shown that IgG antibodies specific to the commensal microbiota are present in blood and may support provide host-microbial homeostasis (58). Our data and others suggest the link between oligosaccharides and immune maturation in early life, which may be mediated in part through changes in the gut microbiome induced by BMOS and HMO (32). In the current study, we demonstrated that incorporating BMOS and HMO into the formula led to immunomodulatory effects in neonatal pigs. The presence of both sources of oligosaccharides resulted in higher concentrations of IgG in the serum, a reduced number of lymphocytes as T-helper cells, and increased secretion of IL-10 and IL-1β in LPS-stimulated PBMC and spleen cells, respectively. This study provides evidence that immune responses are oligosaccharide-specific and combining both neutral and acidic oligosaccharides influence both the adaptive and innate arms of the immune system.

In summary, the commercial availability of HMO has led to the initial supplementation of infant formula with neutral and fucosylated oligosaccharides (44). However, acidic oligosaccharides also provide cognitive and immunological benefits to infants (45). BMOS can be a suitable source of sialylated oligosaccharides that resemble those in human milk (35–37). Taken together, the findings of this study and previously reported outcomes from the same experiment (31, 32, 59) demonstrate the multifactorial actions of HMO and suggest that different oligosaccharides structures have specific activities related to cognitive, microbiome and immune outcomes. Thus, formula-fed infants could benefit from receiving mixtures of HMO in doses similar to those present in human milk on multiple outcomes. Recent randomized controlled trials have shown that mixtures of 5 to 6 oligosaccharides are safe and well tolerated (60–62).

## Data availability statement

The original contributions presented in the study are included in the article/**Supplementary Material**, further inquiries can be directed to the corresponding author.

## Ethics statement

The animal study was approved by University of Illinois at Urbana-Champaign Institutional Animal Care and Use Committee. The study was conducted in accordance with the local legislation and institutional requirements.

## Author contributions

MM: Formal analysis, Investigation, Methodology, Writing – original draft, Writing – review & editing. MW: Formal analysis, Investigation, Methodology, Writing – review & editing. JH: Conceptualization, Writing – review & editing. JY: Conceptualization, Writing – review & editing. RD: Conceptualization, Formal analysis, Funding acquisition, Methodology, Supervision, Writing – review & editing. SD: Conceptualization, Formal Analysis, Funding acquisition, Methodology, Supervision, Writing – review & editing.
