# Supplementary material for: Formula supplementation with human and bovine milk oligosaccharides modulates blood IgG and T-helper cell populations, and ex vivo LPS-stimulated cytokine production in a neonatal preclinical model
Source: Front Immunol. 2023 Dec 20;14:1327853. doi: 10.3389/fimmu.2023.1327853 (PMC10765566; doi:10.3389/fimmu.2023.1327853)
Supplement: Supplementary file 1 [file Table_1.docx]

Supplementary Material

**Formula supplementation with human and bovine milk oligosaccharides modulates blood IgG and T-helper cell populations, and *ex vivo* LPS-stimulated cytokine production in a neonatal preclinical model**

**Marcia H. Monaco, Mei Wang, Jonas Hauser, Jian Yan, Ryan N. Dilger, Sharon M. Donovan***

*** Correspondence:** Sharon Donovan: [sdonovan@illinois.edu](mailto:sdonovan@illinois.edu)

**Supplementary Table 1**. Circulating concentrations of plasma IL-8 and serum LPS binding protein (LPSbp) and Major Acute Phase Protein (MAP) in 33-day-old pigs fed milk replacer with or without BMO, HMO or a combination of both.

| **Cytokine** | **CON** | **HMO** | **BMOS** | **BMOS+HMO** | **Statistics** |
| --- | --- | --- | --- | --- | --- |
| IL-8 (pg/mL) | 11.2 ± 6.3 | 9.1 ± 2.3 | 17.7 ± 4.2 | 8.2 ± 1.7 | N.S. |
| LPSbp (ng/mL) | 52.4 ± 6.0 | 47.3 ± 6.3 | 41.0 ± 4.2 | 44.6 ± 5.6 | N.S. |
| MAP (ng/mL) | 0.46 ± 0.05 | 0.49 ± 0.06 | 0.49 ±0.05 | 0.48 ± 0.05 | N.S. |

Data are presented as mean ± SEM.

N.S. – not significant.

**Supplementary Table 2**. Cytokine concentrations in conditioned media of unstimulated and LPS-stimulated spleen cells from 33-day-old pigs fed milk replacer with or without BMO, HMO or a combination of both.

| **Cytokine** | **Condition** | **CON** | **HMO** | **BMOS** | **BMOS+HMO** | **Statistics** |
| --- | --- | --- | --- | --- | --- | --- |
| IL-1β (pg/mL) | LPS | 392 ± 71 | 352 ± 80 | 937 ± 443 | 1,213 ± 426 | BMOS: p=0.03 |
|  | UNST | 7.4 | 7.4 | 7.4 | 7.4 |  |
|  | Fold-change† | 53 | 48 | 127 | 164 |  |
| IL-10 (pg/mL) | LPS | 19.8 ± 6.1 | 8.3 ± 2.7 | 23.2 ± 12.6 | 31.4 ± 11.5 | N.S. |
|  | UNST | 1.15 | 1.15 | 1.15 | 1.15 |  |
|  | Fold-change | 17 | 7 | 20 | 27 |  |
| TNF-α (pg/mL) | LPS | 290 ± 73 | 184 ± 38 | 310 ± 76 | 496 ± 138 | N.S |
|  | UNST | 18.7 ± 11.4 | 8.8 ± 6.4 | 67 ± 47 | 49 ± 25 |  |
|  | Fold-change | 16 | 21 | 5 | 10 |  |

Data are expressed as means ± SEM. Number of pig samples varied from 6-12.

Mean values with unlike letters were significantly different (p<0.05). Statistical analysis was not conducted on unstimulated values due to non-normal data distribution.

†Fold-change was calculated by dividing the mean cytokine concentration in media of LPS-stimulated cells by the mean concentration secreted by unstimulated cell within each treatment group. If values were below the level of detection, a value reprenting ½ the lowest concentration on the standard curve was used: IL-1β (22.3 pg/mL); IL-10 (3.5 pg/ml); TNF-α (7.0 pg/ml); and IL-8 (31.33 pg/ml) for statistical analyses.

Abbreviations: N.S. – not significant; UNST: unstimulated; fold change calculated by the mean LPS concentration divided by mean unstimulated concentration.
